# Radiogenomics and the DNA damage response: opportunities for biomarker-guided radiosensitization in pancreatic cancer

**DOI:** 10.3389/fonc.2026.1762867

**Published:** 2026-06-08

**Authors:** Marie Nour Karam, Joseph Hajj, Christopher H. Crane, Paul Ramia, Fady Geara, Nadim Hamadeh, Jacob G. Scott, Stephen Grobmyer, Rachael Almond, Eileen M. O’Reilly, Carla Hajj

**Affiliations:** 1 Department of Medicine, Indiana University School of Medicine, Indianapolis, IN, United States; 2 Heart, Vascular, and Thoracic Institute, Cleveland Clinic Foundation, Cleveland, OH, United States; 3 Department of Radiation Oncology, Memorial Sloan Kettering Cancer Center, New York, NY, United States; 4 Department of Radiation Oncology, Cleveland Clinic, Abu Dhabi, United Arab Emirates; 5 Faculty of Medicine, American University of Beirut, Beirut, Lebanon; 6 Department of Radiation Oncology, Cleveland Clinic Foundation, Cleveland, OH, United States; 7 M42, Abu Dhabi, United Arab Emirates; 8 Department of Medical Oncology, Memorial Sloan Kettering Cancer Center, New York, NY, United States

**Keywords:** DNA damage response, genomic-adjusted radiation dose (GARD), KRAS G12D, pancreatic ductal adenocarcinoma, PARP inhibitors, radiogenomics, radiosensitization

## Abstract

Pancreatic ductal adenocarcinoma (PDAC) is associated with poor prognosis, despite significant research efforts. The use of radiotherapy (RT) in PDAC treatment is constrained by intrinsic PDAC radioresistance and concern for normal tissue toxicity. Radiogenomics, the study of how genetic variants may influence RT response, could be incorporated into PDAC treatment. This approach offers a framework for personalized RT dosing guided by individual genomic data. Additionally, DNA damage response (DDR) inhibitors may sensitize PDAC to ionizing radiation to increase the targeted effectiveness of RT. This review examines the history and progress of radiogenomics, spanning from genome-wide approaches to more recent algorithm-based indices such as the radiosensitivity index (RSI) and the genomic-adjusted radiation dose (GARD). These approaches are promising but require additional validation in PDAC due to inconsistent findings across studies and inadequate PDAC-specific data. We also evaluate clinical advances in the development of inhibitors targeting key DDR elements such as ATR, ATM, CHK1/2, WEE1, DNA-PKcs, and PARP. Finally, we highlight emerging strategies for targeting KRAS G12D in PDAC. Collectively, these approaches offer an avenue for reducing radioresistance in PDAC while improving treatment response and minimizing normal tissue toxicity. Future research directions should include the incorporation of multi-omics data into predictive models for appropriate treatment selection, additional large-scale, biomarker-driven clinical trials, and continued integration of biomarker-targeting drug therapy, radiotherapy, and immunotherapy into treatment regimens.

## Key Points:

- Radiogenomics is the study of how individual genetic variation influences response to radiotherapy.
- The radiosensitivity index (RSI) and genomic-adjusted radiation dose (GARD) are radiogenomic indices that may serve as tools for personalized radiotherapy dosing.
- Pancreatic ductal adenocarcinoma (PDAC) is one of the most radioresistant malignancies.
- DNA damage response (DDR) inhibitors may sensitize PDAC to radiotherapy and improve PDAC outcomes.
- KRAS inhibitors represent a novel biomarker-driven opportunity for further PDAC radiosensitization.

## Introduction

Radiation therapy is indicated in the treatment of an estimated 50% of all cancers and is often considered the most efficacious non-surgical cancer therapy (1–4). Radiation therapy targets cancer by creating ions that deliver energy to cells and tissues, causing genetic damage. Normal cells’ DNA and cell repair pathways function to repair this damage, while cancer cells are generally more susceptible, thereby leading to selective cancer cell death (5–7). However, this selectivity is incomplete, and normal tissue toxicity must be considered when selecting a radiation therapy regimen. Herein lies the classic dilemma: choosing a regimen intense enough to kill the cancer cells while preserving normal tissue and avoiding significant patient morbidity and mortality (2–4, 8). Newer radiotherapy (RT) strategies, such as intensity-modulated radiotherapy and stereotactic body radiotherapy, partially circumvent this by sparing healthy tissue while precisely targeting tumors, thereby increasing the therapeutic index (9–11). More recently, adaptive RT platforms (i.e., Ethos, MR-Linac) have enabled real-time treatment adjustments based on daily anatomical changes, further improving accuracy and sparing of organs at risk (12, 13). In parallel, particle therapies, including proton and carbon ion RT, offer distinct physical and biological advantages, expanding the therapeutic window for challenging and radioresistant tumors (14). Yet technical precision alone cannot overcome biologic heterogeneity; inter-patient genetic variation remains a major driver of toxicity and response, motivating the field of radiogenomics.

While technological advances have improved precision in dose delivery, genetic differences between patients remain a critical determinant of both normal tissue toxicity and tumor response, an area of study known as radiogenomics. In this review, radiogenomics refers to the study of inherited genetic differences that affect both normal tissue toxicity and tumor radiosensitivity (2, 3, 8). This is distinct from the imaging definition of radiogenomics, often called “radiogenomic mapping” (15, 16).

Through radiogenomics, further progress may be achieved by allowing dose escalation while simultaneously protecting healthy tissue. A substantial proportion of inter-patient variability in normal tissue radiosensitivity is thought to be heritable and largely determined by biological rather than random factors (3, 4, 8). By leveraging these determinants, radiogenomics holds the potential to improve the therapeutic ratio of RT, enhancing tumor control while reducing treatment-related morbidity.

The purpose of this review is to examine the current literature on radiogenomics in pancreatic cancer and explore potential radiosensitizer drugs that may be paired with radiation therapy to target genetic effectors in pancreatic ductal adenocarcinoma (PDAC). The central premise of our paper is that overcoming radioresistance in PDAC may benefit from a multi-pronged, biomarker-driven approach that combines personalized, radiation-dose determination through radiogenomics, targeted radiosensitization via DDR pathway inhibition, and inhibition of core oncogenes such as KRAS. Together, these may converge into a strategy for improving radiation response in PDAC using precision medicine approaches.

## Radiogenomics of normal tissue toxicity: from SNPs to genome-wide discovery

Radiogenomics began with candidate gene studies investigating single-nucleotide polymorphisms (SNPs) and their association with RT toxicity across malignancies. In these studies, several of the most frequently examined genes included XRCC1, TGF-β, GSTP1, and ATM (2, 3, 8, 17). Most of these studies were underpowered, producing inconsistent results (8, 17). Several tested multiple hypotheses at once, which raised concern for the multiple comparisons problem (8, 17).

The field has since progressed to genome-wide discovery approaches, including genome-wide association studies (GWAS), which survey the entire genome for variants linked to radiation toxicity, uncovering novel associations beyond traditional radiation biology pathways. GWAS studies compare genotypes to identify sites of linkage disequilibrium that could account for observed phenotypic differences (18, 19). Early GWAS identified SNP associations with erectile dysfunction (20, 21), urinary frequency (22), urinary stream (22), rectal bleeding (23), overall toxicity following prostate cancer RT (24, 25), mucositis (26), and complications after RT for breast cancer, such as lymphedema or nipple retraction (27). However, GWAS also share limitations, including the “winner’s curse” phenomenon (8). GWAS-positive “hits” that achieve statistical significance usually involve an entire region of the genome consisting of many SNPs in series, which can be difficult to interpret, particularly because up to 88% of SNPs detected in GWAS are in non-coding segments (28). Complex, reciprocal gene-gene interactions also limit the predictive power of simple association studies (2). Given these limitations, predictive composite models were developed to interpret genomic evidence into clinically actionable treatment guidance.

## Radiosensitivity index (RSI) and genomic adjusted radiation dose (GARD): toward precision radiation oncology

Predictive algorithms represent a major development in radiogenomics, led by the radiosensitivity index (RSI) and later, the genomic-adjusted radiation dose (GARD). The RSI, developed by Eschrich et al. in 2009 (29), served as a discovery platform for biomarkers that could predict radiation response. The initial discovery set used 48 cancer cell lines with known survival fraction at 2 Gy (SF2) to identify 10 “hub” genes associated with radiation sensitivity. Accordingly, the researchers developed a linear regression model to predict SF2, which achieved statistical significance once leukemia cell lines were excluded. In a parallel paper, the RSI successfully predicted responders to RT in rectal, esophageal, and head and neck cancers (30). This model was later validated in glioblastoma (31), breast (32, 33), pancreatic (discussed further below) (34), endometrial (35), and prostate cancers (36).

The GARD combines the RSI, the standard radiation dose, and the standard fractionation schedule via the linear-quadratic model to predict clinical outcomes from ionizing radiation (37, 38). Higher GARD scores suggest radiosensitivity, whereas lower GARD scores suggest radioresistance. RSI provides a genomic measure of a tumor’s radiosensitivity, whereas GARD integrates this radiosensitivity with the prescribed physical dose to estimate the actual biological effect of treatment. In practice, RSI identifies which tumors are sensitive, and GARD translates that information into a patient-specific prediction of therapeutic impact, guiding personalized RT dosing and possible need for dose escalation. While gliomas and sarcomas had the lowest median GARD scores, indicating radioresistance, PDAC had the lowest GARD scores when modeled at a standardized regimen of 70 Gy delivered in 35–40 fractions (39). Subsequent validation studies of GARD showed it to be an independent predictor of local control in triple-negative breast cancer patients after breast conservation surgery and RT (38). In a pooled analysis of 7 cancer types, GARD predicted overall survival (OS) and time to first recurrence (40). More recently, in an analysis of HPV-positive oropharyngeal squamous cell carcinoma, de-escalating RT doses on a personalized basis according to a patient’s unique GARD score was modeled to maintain the same overall cure rates at the population level (41). This contrasts with the recent phase II/III clinical trial HN005, showing that uniform dose de-escalation in this demographic was associated with inferior outcomes (41, 42).

The most recent extension of GARD is RxRSI, which represents the *personalized radiation dose* a patient would need, given their tumor’s radiosensitivity, to reach a biologically effective threshold (GARD ≥ 33) associated with better tumor control. In non-small cell lung cancer, only 25% of patients achieved RxRSI, the modeled optimal individualized dose, using standard-of-care doses. Some patients may be underdosed, risking disease persistence or recurrence, whereas others may be overdosed, risking toxicity (43).

GARD has been recognized by *The Lancet Oncology* as a “future cancer research priority” (43, 44). Additionally, the European Organization for Research and Treatment of Cancer acknowledged the evidence in favor of the RSI/GARD system and endorsed eventual entry into clinical trials (43, 45). However, Mistry and colleagues have challenged the validity of RSI and GARD, citing weak correlation with experimental measures of radiosensitivity, arbitrary categorization of RSI in study analyses, and limited evidence that GARD explains more variability in outcomes than its constituents, such as RSI or dose alone (46).

## Unraveling radioresistance in pancreatic cancer through radiogenomics

PDAC constitutes 90% of all pancreatic malignancies and is the third most common cause of cancer deaths in the U.S., despite being only the 10^th^ - 11^th^ most prevalent malignancy (47). This is in part due to most diagnoses occurring at an advanced stage, with vascular involvement already present (47, 48). Currently, the 5-year relative survival is 13%, a modest improvement compared to the 5-year survival of approximately 4% in the 1990s (49).

To date, there are no GWAS that examine radiation resistance in PDAC. A single-candidate-gene study by Zeng et al. (50) identified 4 SNPs that may modify RT response in PDAC. SNPs in the RRM1 gene (encoding a ribonucleotide reductase subunit) were the most notable; in patients who had the *RRM1* rs1662172 AG (heterozygous adenosine and guanine) or GG (homozygous guanine) genotype and underwent RT, the hazard ratio was 2.18 when compared to those with the AA genotype (p = 0.00063).

The RSI has been tested in PDAC by Strom et al. (34). Patients with local resectable PDAC were stratified into a radiosensitive group and a radioresistant group based on RSI. Multivariate analysis yielded a worse OS in radioresistant, high-RSI tumors; however, this effect was not quite statistically significant (p = 0.054). In those treated with RT with high-risk disease, patients with low-RSI scores had longer OS than those with high-RSI scores (31.2 vs. 13.2 months, p = 0.04). RT dose did not predict OS (34).

Later, GARD was studied in PDAC using the Moffitt Cancer Center pancreatic cancer cohort (n = 40, postoperative RT dose range 43.2–54 Gy in 24–30 fractions) (39). Within the 45 Gy dose range analysis, the pancreatic cancer cohort exhibited the lowest median GARD score of all malignancy types, reflecting intrinsic radioresistance. GARD correlated with OS after adjustment for CA 19–9 and margin lymph node status (hazard ratio 2.6, CI 1.1 - 6.0; p = 0.029), though dose alone did not predict benefit (39), underscoring the importance of incorporating genomics into treatment planning. However, a subsequent study investigated GARD for 11 clinical cohorts, including PDAC. Here, GARD showed an association with OS and time to first recurrence, but in an individual pancreatic cancer cohort analysis, there was no association with OS (40) (hazard ratio = 1.00, 0.94 - 1.06).

There are several clinically important considerations in evaluating the utility of RSI and GARD, particularly in PDAC. Firstly, the PDAC microenvironment is characterized by a highly desmoplastic stroma (51); accordingly, it remains unclear whether the bulk-tumor transcriptomic data used to train the RSI truly reflect intrinsic cell radiosensitivity, rather than the stromal admixture (52). Similarly, this model assumes uniform radiosensitivity across all patients of a certain tumor type; however, evidence suggests that other conditions in the microenvironment of a tumor (such as hypoxia or nutrient availability) impact radiosensitivity just as intrinsic gene expression patterns do (37). Additionally, there are concerns about the applicability of the linear-quadratic model to predict radiosensitivity in high-dose, hypofractionated regimens, which are now more frequently being used in PDAC (53).

As previously indicated, another glaring concern in studies of GARD in PDAC is the discrepancy of findings between the two seminal studies, as one study found statistically significant associations with GARD and OS after adjustment for CA 19–9 and node status (39), while the multi-cohort study did not reproduce this finding in PDAC-specific analyses (40). There are several plausible sources for this discrepancy, including (i) small cohort sizes, (ii) lack of standardized treatment exposures, and (iii) inappropriate endpoint selection, as metastasis dominates even resectable PDAC cases, such that the endpoint of OS may be too difficult to interpret, and a more proximal endpoint, such as local control, may be more suitable (54).

Future research endeavors should aim to validate radiogenomic predictors with large-scale studies that address these considerations by prioritizing uniform treatment regimens, larger cohort sizes, appropriate endpoint selection, and evaluations of tumor purity. This could improve survival outcomes, reduce treatment toxicity, and guide the transition from empirical radiation dosing to precision-based regimens.

## Overcoming radioresistance in pancreatic cancer: targeting DNA damage response pathways

To understand radioresistance in pancreatic cancer, the normal cellular response to radiation must first be discussed. Upon irradiation, DNA damage in cells can trigger one of several outcomes, such as cell-cycle arrest, or, if the damage is too extensive, then mitotic catastrophe, apoptosis, senescence, necrosis, or autophagy may occur (55). Targeting DNA damage response (DDR) proteins can impair cancer cells’ ability to repair DNA to enhance cell death and increase tumor radiosensitivity. This section outlines the key DDR pathways and current evidence for targeted radiosensitizer strategies in PDAC.

On average, PDAC harbors 63 gene mutations (56). At least part of the clinical variation in radiation response may be attributed to such mutations. For example, KRAS is mutated in up to 90% of PDAC cases (57), and studies indicate that KRAS-activating mutations, such as KRAS G12D, contribute to radiation resistance (58). The DDR pathway detects DNA damage and propagates downstream events such as halting the cell cycle, initiating DNA repair, activating apoptosis, and other functions (59). Disruption of this cellular pathway by genetic mutations, such as alterations in KRAS, ATM, ATR, and other genes, allows for the evasion of apoptosis, the acceleration of DNA damage, and the accrual of mutations (60). Paradoxically, DDR inhibition can radiosensitize tumors by inducing mitotic catastrophe and death (61). In theory, the combination of RT with a radiosensitizer may augment treatment response, improve remission rates, and improve survival, particularly in PDAC, which is highly resistant to radiation (59). These compelling hypotheses have prompted investigation into DDR pathway inhibition, with trials testing PARP, ATR, ATM, DNA-PK, and WEE1 inhibitors.

## Molecular targets in PDAC: DNA damage response pathways

The DDR is a complex network that orchestrates specialized repair processes depending on the nature of DNA damage incurred. Key players of this pathway responding to double-stranded breaks (DSBs) include the ATM and ATR kinases. At the most basic level, ATM is recruited to sites of double-stranded DNA (dsDNA) damage and activates CHK2. CHK2 promotes the stabilization of p53 and the transcription of p21, leading to cell cycle arrest and/or other cell cycle alterations at the G1/S checkpoint (60, 62). ATR is recruited to sites of replication stress, such as a stalled replication fork, to activate CHK1, phosphorylating targets such as WEE1. WEE1 inhibits cyclin-dependent kinases, preventing mitosis (60, 62). It is important to note that ATM and ATR are now known to have hundreds of targets with more complex effects than solely what is stated above (62). PARP1 and PARP2, in contrast, detect single-strand DNA breaks (SSBs) and catalyze the poly(ADP-ribosyl)ation (PARylation) to substrates that recruit scaffold proteins and ligases to repair the breaks (62, 63).

**Figure 1:**
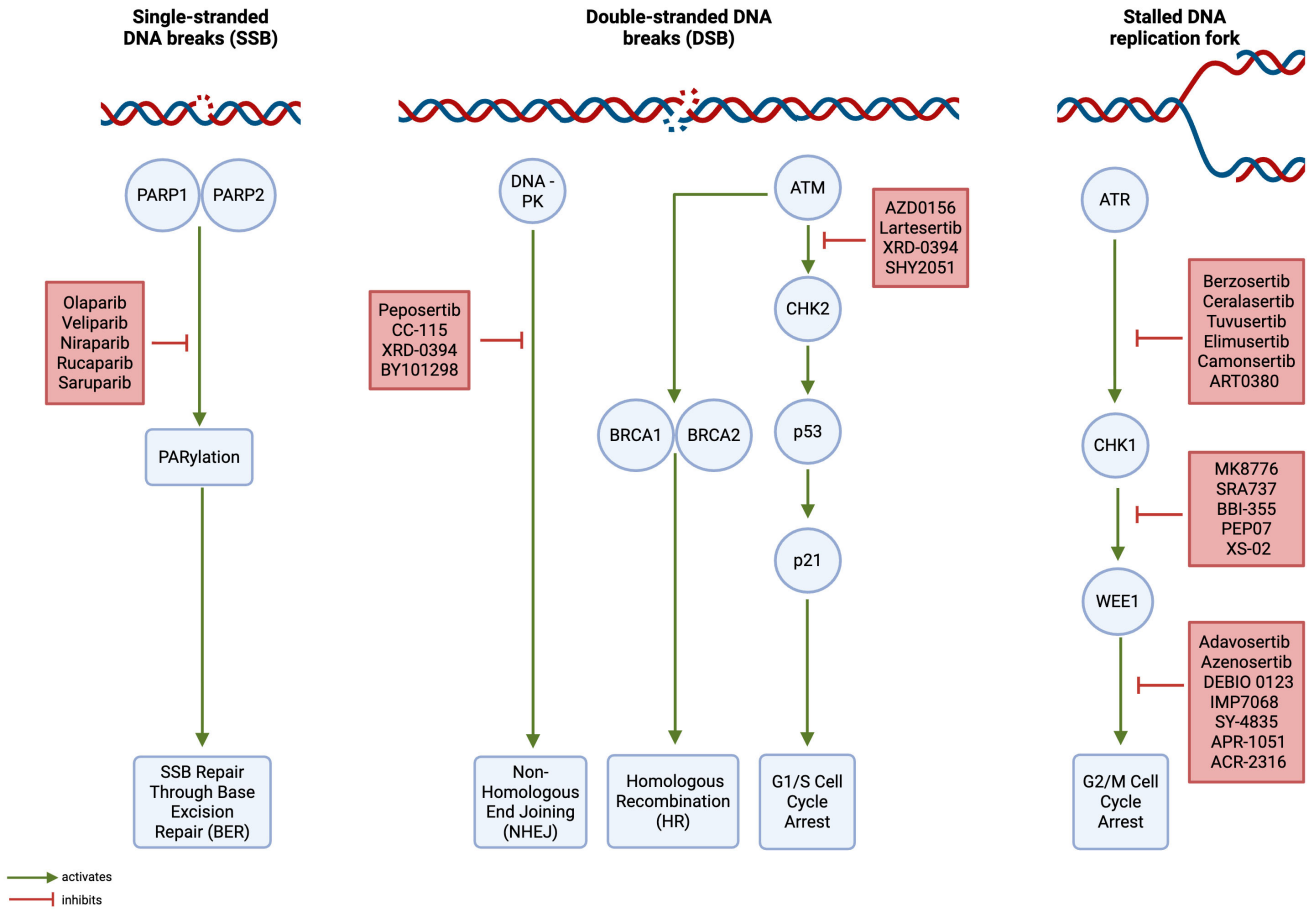
Simplified overview of key DNA damage response (DDR) effectors and their investigational drug inhibitors. Olaparib, veliparib, niraparib, rucaparib, saruparib = PARP inhibitors; peposertib, CC-115, XRD-0394, BY101298 = DNA PK inhibitors; AZD0156, lartesertib, XRD-0394, SHY2051 = ATM inhibitors; berzosertib, ceralasertib, tuvusertib, elimusertib, camonsertib, and ART0380 = ATR inhibitors; MK8776, SRA737, BBI-355, PEP07, and XS02 = CHK1 inhibitors; adavosertib, azenosertib, DEBIO 0123, IMP7068, SY-4835, APR-1051, and ACR-2316 = WEE1 inhibitors. SSB = single-stranded break; DSB = double-stranded break; PARylation = addition of poly(ADP-ribose) chains to proteins by PARP enzymes. BER = base-excision repair; NHEJ = non-homologous end joining; HR = homologous recombination. Created in BioRender. Karam, M (2026). https://BioRender.com/l9x1peg.

Once DNA damage is detected, different DNA repair pathways may be initiated. DSBs can be repaired by the high-integrity method, homologous recombination (HR), or the low-integrity method, non-homologous end joining (NHEJ). Alterations to the DNA backbone or the DNA bases that do not create single-stranded DNA (ssDNA) nick can be repaired through base excision repair, mismatch repair, and nucleotide excision repair pathways (60, 63). A simplified schematic of the DDR pathway and representative inhibitors under study is displayed in **Figure 1**. Importantly, though inducing synthetic lethality via DDR inhibition is a mechanistically promising approach, at this time only PARP inhibition has been sufficiently researched to justify its use in the clinical setting. The remaining DDR nodes discussed in this section, including WEE1 inhibitors, ATR inhibitors, ATM inhibitors, CHK1 inhibitors, and DNA-PK inhibitors, are still in the exploratory stages of clinical investigation.

Another major consideration in DDR inhibition-radiotherapy combination strategies is toxicity. By bypassing cell-cycle checkpoints and inducing mitotic catastrophe, DDR inhibitors not only impact cancer cells but also result in normal tissue toxicity, notably myelosuppression. In PDAC specifically, local RT regimens are already limited by the pancreas’s proximity to luminal organs, resulting in GI toxicity (64). As previously mentioned, PARP inhibitors have the strongest clinical signal thus far but are not without side effects. In the seminal POLO trial of PARP inhibitor olaparib (discussed further in the next section), 40% experienced a grade 3 or higher adverse event, compared to 23% in the placebo group, with the most common serious adverse effect being anemia (65) (NCT02184195). Future efforts require careful dose optimization and patient selection to maximize efficacy while also ensuring an acceptable therapeutic index.

### PARP inhibitors

As previously mentioned, PARP enzymes play an important role in SSB repair. PARP inhibitors are indicated in the treatment of malignancies that are BRCA1/BRCA2-deficient by inducing synthetic lethality. Overall, 5 - 10% of familial PDAC and 3% of sporadic PDAC are characterized by a germline BRCA1/2 mutation (66). Given that BRCA1/BRCA2 play important roles in HR, it was posited that the inhibition of PARP causes replication fork collapse, producing DSBs that are irreparable in BRCA1/2-deficient individuals (62).

The landmark POLO phase III clinical trial (65) (NCT02184195) assessed the PARP inhibitor olaparib as maintenance therapy in metastatic PDAC with germline BRCA1 or BRCA2 mutations and stable disease after prior platinum-based therapy (such as FOLFIRINOX (folinic acid, fluorouracil (5-FU), irinotecan, oxaliplatin). A statistically significant difference in progression-free survival (PFS), the primary endpoint, was observed (7.4 months vs. 3.8 months; hazard ratio 0.51; 95% CI 0.34 - 0.78). However, at the time of primary endpoint cutoff, there was no statistically significant difference in OS (65) (18.9 months vs. 18.1 months; hazard ratio 0.91; 95% CI 0.56 - 1.46). An updated survival analysis in 2020 confirmed the lack of OS benefit but did show significant differences in secondary endpoints such as interval to subsequent cancer therapies or death, and time to stopping study treatment or death (67). This trial led to FDA approval of olaparib for the maintenance therapy of stable, metastatic PDAC in this patient population (68).

Olaparib remains the only FDA-approved PARP inhibitor for PDAC. Studies of olaparib in other PDAC demographics and in combination with other therapies are under investigation. Two now-completed phase II trials of olaparib in PDAC with “BRCAness” features (BRCA wild-type but with DDR mutations) showed preliminary findings of stable disease and partial response in platinum-sensitive patients only (69) (NCT02511223, NCT02677038). A phase I trial of olaparib and gemcitabine was safely completed but did not show differences in efficacy from gemcitabine alone. However, the patients in this cohort were not selected for genetic HR defects, which could account for this finding (70) (NCT00515866). Alternative PARP inhibitors are also under study; in a now-completed phase Ib/II trial, the PARP inhibitor niraparib with the anti-PD-1 antibody nivolumab showed a favorable 6-month PFS, whereas the 6-month PFS of niraparib with the anti-CTLA-4 antibody ipilimumab was nonsignificant (71) (NCT03404960). Finally, the PARP inhibitor rucaparib was evaluated in the phase II RUCAPANC study as a monotherapy in pretreated locally advanced or metastatic PDAC with either germline or somatic BRCA mutations. The study was halted after the interim analysis due to not meeting the predetermined primary endpoints; however, the disease control rate was 31.6% (72) (NCT02042378).

BRCA2-RAD51 disruption is an emerging strategy that may one day extend the utility of PARP inhibitors beyond BRCA-mutated cancers. For HR to proceed, BRCA2 mediates recruitment of RAD51 to bind dsDNA, detect homologous segments, and enable strand exchange (73). Thus, blocking the BRCA2-RAD51 interaction would mimic BRCA2 deficiency, inhibit HR, and induce susceptibility to PARP inhibitors. *In vitro* research has shown small organic molecules, DNA aptamers, and chimeric proteins that induce synthetic lethality in pancreatic cancer cells and sensitize them to the PARP inhibitor olaparib, similar to the effects seen in patients with BRCA1/2 mutations (74–80). Importantly, this approach exploits the concept of “between-pathway” synthetic lethality, in which multiple nodes across separate but interconnected DNA repair pathways are targeted to induce cell death. However, it may also be possible to exploit “within-pathway” synthetic lethality by simultaneously targeting multiple nodes within the same repair pathway. This concept was demonstrated by Masi et al., in which the BRCA2-RAD51 inhibitor RS-35d and its enantiomers not only disrupted the BRCA2-RAD51 interaction but also inhibited ATM, ATR, and DNA-PK, ultimately overcoming PARP inhibitor resistance in BRCA2-deficient PDAC models (81). Though this research remains preclinical, it is a plausible future pathway for biomarker-targeted treatment in PDAC.

RAD52 also merits brief mention in the context of PARP-mediated tumor killing. Though canonical HR relies on the BRCA1/2-dependent pathway, RAD52 has emerged as a mediator of “backup” homology-driven repair in BRCA1/2-deficient models and under other conditions of replication stress (82). From a therapeutic standpoint, this implies that RAD52 inhibition may abrogate any residual HR occurring in BRCA2-deficient, PARP inhibitor-treated models to potentially augment antitumor effects. Moreover, RAD52 may be a suitable target for overcoming PARP inhibitor resistance, as recent research suggests (83). Though the evidence remains preclinical, RAD52 is a reasonable target that warrants future investigation.

Currently, the PARP inhibitor olaparib in BRCA-negative PDAC is an excellent example of the potential for DDR inhibitors to serve as radiosensitizers. Preclinical evidence supports the role of olaparib as a radiosensitizer in PDAC (84, 85), and an active clinical trial (NCT05411094) is evaluating its combination with RT in patients with locally advanced, unresectable PDAC. Ongoing studies include maintenance, combination, and RT-containing designs, which are summarized in **Table 1**.

**Table 1:** Ongoing clinical trials of PARP inhibitors in PDAC.

Trial ID	Sponsor	Phase	Status	Intervention
NCT02890355	NCI	II	active, not recruiting	veliparib + mFOLFIRI vs. FOLFIRI in mPDAC
NCT03337087	Academic and Community Cancer Research United	I/II	active, not recruiting	NaI-IRI, FU, leucovorin calcium, and rucaparib in mPDAC*
NCT04348045 (MAZEPPA)	GERCOR - Multidisciplinary Oncology Cooperative Group	II	active, not recruiting	olaparib in mPDAC with BRCAness phenotype (Arm A) selumetinib + durvalumab (Arm B) or FOLFIRI alone (Arm C) in mPDAC with KRAS mutation
NCT05659914	Spanish Cooperative Group for the Treatment of Digestive Tumors (TTD)	II	active, not recruiting	olaparib + durvalumab in DDR- mutated mPDAC
NCT01585805	NCI	II	active, not recruiting	Part 1: gemcitabine + cisplatin ± veliparib in advanced PDAC with BRCA1/2 or PALB2 mutation Part 2: veliparib monotherapy in advanced pretreated PDAC
NCT03601923	Dana-Farber Cancer Institute	II	active, not recruiting	niraparib in advanced PDAC with DDR mutation
NCT03553004 (NIRA-PANC)	University of Kansas Medical Center	II	unknown status	niraparib in pretreated mPDAC with DDR mutations
NCT04548752	NCI	II	recruiting	olaparib vs. POLAR in gBRCAmt mPDAC
NCT04753879	Sidney Kimmel Comprehensive Cancer Center at Johns Hopkins	II	recruiting	GAX-CI followed by POLAR in mPDAC
NCT04666740	Memorial Sloan Kettering Cancer Center	II	active, not recruiting	POLAR in mPDAC with platinum sensitivity or HR deficiency
NCT05093231	Cambridge University Hospitals NHS Foundation Trust	II	recruiting	POLAR in mPDAC with high mutational load or MMR deficiency
NCT04005690	OHSU Knight Cancer Institute	Early Phase I	recruiting	olaparib, tremelimumab, saruparib, azenosertib, onvansertib, or cobimetinib in PDAC
NCT04858334 (APOLLO)	NCI	II	recruiting	olaparib vs. placebo in PDAC with BRCA1/2 or PALB2 mutation that has been resected and treated with chemotherapy with curative intent
NCT05257993	Onconic Therapeutics Inc.	I	recruiting	JPI-547 with mFOLFIRINOX or with Gemcitabine/nab-paclitaxel advanced PDAC
NCT05411094	NCI	I	recruiting	olaparib + durvalumab + RT in pretreated unresectable PDAC
NCT06078787	Azienda Ospedaliero-Universitaria di Modena	II	recruiting	olaparib in pretreated and progressive locally advanced or metastatic PDAC with PALB2 mutation
NCT06747845 (ParpVax2)	Abramson Cancer Center at Penn Medicine	II	recruiting	maintenance niraparib + ipilimumab in stable mPDAC after platinum-based regimen

NCI, National Cancer Institute; Olaparib, veliparib, niraparib, saruparib, PARP inhibitors; FOLFIRI, 5-fluoruracil (5-FU), leucovorin, and irinotecan; mFOLFIRI, modified FOLFIRI, differing by absence of day 1 5-FU bolus; NaI-IRI, nanoliposomal irinotecan; FU, fluorouracil; mPDAC, metastatic PDAC; BRCAness, Homologous recombination-deficient malignancy without BRCA1/2 mutation; selumetinib, cobimetinib, MEK1/2 inhibitors; durvalumab, anti-PD-L1 monoclonal antibody; DDR, DNA Damage Response; gBRCAmt, germline BRCA1/2 mutation; POLAR, pembrolizumab + olaparib; GAX-CI, gemcitabine, nab-paclitaxel, capecitabine, cisplatin, and irinotecan; MMR, mismatch repair; pembrolizumab, anti-PD-1 monoclonal antibody; tremelimumab, ipilimumab, anti-CTLA-4 monoclonal antibody; azenosertib, Wee1 inhibitor; onvansertib, PLK1 inhibitor; JPI-547, combined PARP and tankyrase inhibitor; nab-paclitaxel, nanoparticle albumin–bound paclitaxel; RT, radiotherapy. *other malignancies were also investigated in this study. **includes patients with other pancreatic lesions or malignancies other than PDAC.

### Wee1 inhibitors

WEE1 is a CHK2-regulated kinase that maintains the G2/M checkpoint by phosphorylating cyclin-dependent kinase 1, inhibiting it, and halting the cell-cycle (86, 87) (**Figure 1**). Studies of the WEE1 inhibitor adavosertib (also known as AZD1775/MZT-1775) in preclinical p53-deficient PDAC models demonstrated synergy with gemcitabine (88). Clinically, phase I and phase Ib trials demonstrated a tolerable safety profile and some antitumor activity (89–91) (NCT01748825, NCT02482311, NCT02610075). In a separate, phase I study of locally advanced PDAC treated with adavosertib, radiation, and gemcitabine, preliminary median OS and PFS were 21.7 and 9.4 months, respectively, which are greater than OS and PFS rates from historical studies utilizing gemcitabine and RT (92) (NCT02037230). Results from completed clinical trials of WEE1 inhibitors are summarized in **Table 2** and **Figure 1**.

**Table 2:** Completed clinical trials of WEE1-inhibiting agents.

Trial ID	Phase	Sponsor	Intervention	Primary endpoint	Result
NCT01748825	I	NCI	adavosertib monotherapy in advanced, refractory solid cancers	-determine PK -safety and tolerability (TEAEs and DLTs)	-MTD established -PRs recorded -acceptable safety profile -target engagement demonstrated (90)
NCT02482311	Ib	AstraZeneca	part A: adavosertib in AST part B: adavosertib monotherapy in biomarker-selected ovarian, TNBC, SCLC	-safety and tolerability (TEAEs and DLTs)	-acceptable safety profile -MTD determined (89)
NCT02610075	Ib	AstraZeneca	adavosertib monotherapy in AST	-safety and tolerability (TEAEs, DLTs) -determine MTD	-MTD established -acceptable safety profile -some antitumor effects (91)
NCT02511795	Ib	AstraZeneca	part A: adavosertib + olaparib in refractory solid cancers part B: adavosertib in previously treated SCLC	-safety and tolerability (TEAEs, DLTs) -determine MTD	-MTD confirmed -acceptable safety profile -minor antitumor activity in Part A (147)
NCT02037230	I/II	University of Michigan Rogel Cancer Center	adavosertib + RT + gemcitabine in locally advanced PDAC	-determine MTD	-median OS: 21.7 months -median PFS: 9.4 months -acceptable safety profile -MTD determined (92)
NCT02341456	Ib	AstraZeneca	adavosertib + carboplatin ± paclitaxel in Asian patients with AST	-safety and tolerability (TEAEs, DLTs)	- RP2D identified -PRs recorded (148)
NCT00648648	I	Merck Sharp & Dohme LLC	part 1: adavosertib part 2: adavosertib + gemcitabine, carboplatin, or cisplatin	-safety and Tolerability (TEAEs, DLTs) -% pCDC2 skin cells at baseline & after treatment -adavosertib plasma concentration after treatment -adavosertib mean urine excretion after treatment	-MTDs established in Part 2 -acceptable safety profile -PR and SD recorded -adequate target engagement (149)

NCI, National Cancer Institute; adavosertib, WEE1 inhibitor (other names: AZD1775, MK-1775); PK, pharmacokinetics; TEAEs, treatment-emergent adverse events; DLT, dose-limiting toxicity; MTD, maximum tolerated dose; PR, partial response; AST, advanced solid tumors; TNBC, triple-negative breast cancer; SCLC, small-cell lung cancer; olaparib, PARP inhibitor; RT, radiotherapy; OS, overall survival; PFS, progression-free survival; RP2D, recommended Phase II dose; %pCDC2, % cells positive for phosphorylated cyclin-dependent kinase 1; SD, stable disease. * NCT03313557 is a completed continuation trial of adavosertib that was excluded due to no published results.

There are additional clinical trials of adavosertib (NCT04439227) and other WEE1 inhibitors, including the agents azenosertib (93) (NCT06015659), DEBIO 0123 (94) (NCT05109975), IMP7068 (NCT04768868), SY-4835 (NCT05291182), APR-1051 (NCT06260514), and ACR-2316 (NCT06667141), though overall, WEE1 inhibition is still investigational (**Figure 1**).

### ATR inhibitors

ATR functions to arrest the cell-cycle at the G2/M checkpoint in situations of replication stress (95, 96) (**Figure 1**). Preclinically, numerous studies demonstrated both chemosensitization and radiosensitization with various ATR inhibitors such as berzosertib and ceralasertib (97–99) (**Figure 1**). Clinically, studies of berzosertib with a PARP inhibitor and cisplatin were deemed safe, with partial clinical responses and stable disease observed (100) (NCT02723864). Berzosertib with gemcitabine also induced stable disease or partial responses (101) (NCT02157792). However, some biomarker-selecting strategies were not successful, as trials testing ATR inhibitors in ATM-deficient tumors did not yield clinical benefit (102, 103) (NCT03718091, NCT04564027). Several other ATR inhibitors are being actively investigated (104, 105) (NCT03188965, NCT04095273) and are described in **Table 3** and **Figure 1**.

**Table 3:** Clinical trials of ATR-targeting drugs in PDAC.

Trial ID	Sponsor	Phase	Status	Intervention
NCT04826341	NCI	I/II	recruiting	sacituzumab govitecan and berzosertib in PARP-resistant cancers with HR-deficiencies
NCT02595931	NCI	I	active, not recruiting	berzosertib + irinotecan in AST
NCT03682289	Rahul Aggarwal	II	recruiting	ceralasertib + durvalumab or + olaparib in AST
NCT03669601(ATriUM)	CCTU-Cancer Theme	I	unknown	ceralasertib + gemcitabine in AST
NCT02223923 (Patriot)	Royal Marsden NHS Foundation Trust	I	active, not recruiting	ceralasertib ± RT in refractory solid tumors
NCT05514132	AstraZeneca	I	active, not recruiting	ceralasertib + durvalumab in AST
NCT04170153 (DDRiver Solid Tumors 301)	EMD Serono Research & Development Institute, Inc.	I	active, not recruiting	tuvusertib in AST
NCT05396833 (DDRiver Solid Tumors 320)	EMD Serono Research & Development Institute, Inc.	Ib	recruiting	tuvusertib + avelumab or lartesertib in AST
NCT05691491	NCI	I/II	recruiting	tuvusertib + temozolomide in AST
NCT04616534	NCI	I	active, not recruiting	elimusertib + gemcitabine in AST
NCT04497116 (TRESR)	Repare Therapeutics	I/IIa	active, not recruiting	camonsertib + talazoparib or + gemcitabine in DDR-mutated AST
NCT04972110 (ATTACC)	Repare Therapeutics	I/II	active, not recruiting	camonsertib + niraparib or + olaparib in DDR mutated AST
NCT04657068	Artios Pharma LTD	I/IIa	recruiting	ART0380 monotherapy or dual therapy with either gemcitabine or irinotecan in AST
NCT04095273	Bayer	Ib	completed*	elimusertib + pembrolizumab in AST
NCT03309150	Merck KGaA, Darmstadt, Germany	I	completed*	continuation trial of berzosertib in AST
NCT05469919	AstraZeneca	I	completed*	ceralasertib in Japanese patients with AST

NCI, National Cancer Institute; berzosertib, ceralasertib, tuvusertib, elimusertib, camonsertib, and ART0380, ATR inhibitors. sacutizumab govetican, antibody-drug conjugate delivering SN-38 chemotherapy to Trop-2-expressing cells. HR, homologous recombination; AST, advanced solid tumors; veliparib, talazoparib, niraparib, and olaparib, PARP inhibitors; durvalumab, avelumab, anti-PD-L1 monoclonal antibodies; RT, radiotherapy; lartesertib, ATM inhibitor; temozolomide, alkylating agent; pembrolizumab, anti-PD-1 monoclonal antibody. * = completed trial without published results.

### ATM inhibitors

ATM mediates the detection and repair of DSBs by halting the cell-cycle at the G1/S checkpoint (60, 62, 63) (**Figure 1**). Thus, inhibition of ATM presumably results in mitotic catastrophe and death of cancer cells when combined with another DNA-damaging treatment, be it RT or chemotherapy.

Of the drug candidates that are being investigated clinically, AZD0156 and Lartesertib (or M4076) have shown chemosensitization and/or radiosensitization in preclinical studies (106–108) and are in the early stages of clinical investigation via phase I trials (109, 110) (NCT02588105, NCT04882917, NCT05396833) (**Figure 1**). Additionally, the novel dual ATM and DNA-PK inhibitor XRD-0394 potentiated RT both *in vitro* and *in vivo*, in fact allowing for lower doses of radiation to achieve the same degree of tumor regression in MDA-MB-231 (human triple-negative breast cancer) xenografts. It also synergized with PARP inhibitor niraparib in the BRCA2-mutated PDAC cell line Capan-1 (111). Now, a phase I trial of XRD-0394 and palliative RT was completed in locally advanced, recurrent, or metastatic cancer (112) (NCT05002140). Finally, the ATM inhibitor SHY2051 is being tested in a phase I/II trial with SYS6010, an antibody-drug conjugate targeting the epidermal growth factor receptor (EGFR) and linked to the topoisomerase I inhibitor JS-1 (NCT06775236) (**Figure 1**).

### CHK1 inhibitors

CHK2 and CHK1 serine/threonine kinases are downstream targets of ATM and ATR, respectively. At the G1/S checkpoint, ATM activates CHK2, which mediates G1/S checkpoint arrest (62, 113). ATR phosphorylates CHK1 to arrest the cell-cycle at the G2/M checkpoint (95, 96) (**Figure 1**).

Several iterations of CHK1 inhibitors exist. The selective CHK1 inhibitor MK8776 was well tolerated in phase I trials of MK8776 monotherapy and demonstrated clinical activity as dual therapy with gemcitabine, even in tumors initially resistant to chemotherapy (114) (NCT00779584). Several CHK1 inhibitors, such as PEP07 and XS-02, are under study in early clinical trials (NCT05983523, NCT06531486) (**Figure 1**). Studies of the CHK1 inhibitor SRA737 (or CCT245737) are also ongoing, but thus far have revealed mixed results of clinical efficacy (115, 116) (NCT02797964, NCT02797977). Most recently, the oral CHK1 inhibitor BBI-355 is an extrachromosomal DNA-directed therapy (ecDTx) being studied in cancers with high copy number amplifications of oncogenes found within extrachromosomal DNA. This property typically confers resistance to even targeted therapies, but at the cost of increased replication stress, making these cancers more susceptible to DDR-inhibiting agents (117). Consequently, preclinical evidence of BBI-355 shows antitumor activity in tumors with extrachromosomal DNA facilitating oncogene amplification, specifically in tandem with therapies that target the upregulated oncogene (118). Phase I/II POTENTIATE trials for BBI-355 are ongoing (118) (NCT05827614) (**Figure 1**).

### DNA-PK inhibitors

DNA-dependent protein kinase (DNA-PK) is a central mediator of NHEJ, the primary process of dsDNA repair for DSBs that occur outside of DNA replication (96, 119). DNA-PK is a holoenzyme that consists of two key constituents: the DNA-PK catalytic subunit and the Ku70/Ku80 heterodimer (96, 120, 121) (**Figure 1**).

When first developed, the DNA-PK inhibitor peposertib (also known as M3814/MSC2490484A) induced complete tumor regression with fractionated RT in mouse xenografts (122, 123) (**Figure 1**). In PDAC, peposertib augmented type I interferon signaling and PD-L1 levels with radiation (124), making it a candidate for immunotherapy potentiation as well. However, clinically, it has yielded inconsistent results, with only one of three clinical trials thus far showing adequate clinical activity (125–127) (NCT02316197, NCT03724890, NCT04172532), though additional studies of peposertib are still active (see **Table 4**). Additional agents under investigation are summarized in **Table 4** and **Figure 1**.

**Table 4:** Ongoing clinical trials of DNA-PK-targeting drugs in PDAC.

Trial ID	Sponsor	Phase	Status	Intervention
NCT05687136	NCI	I	recruiting	peposertib + tuvusertib in AST
NCT05868174	Telix Pharmaceuticals (Innovations) Pty Limited	I	recruiting	peposertib + 177Lu-TLX250 I in CAIX-positive AST
NCT04172532	NCI	I/II	recruiting	peposertib + RT in locally advanced PDAC
NCT04068194	NCI	I/II	recruiting	phase I: peposertib + avelumab + RT in AST phase II: RT + avelumab ± peposertib only in advanced gallbladder cancer or cholangiocarcinoma
NCT06462716	Chengdu Baiyu Pharmaceutical Co., Ltd.		active, not recruiting	BY101298 in AST
NCT05002140	XRad Therapeutics Inc	I	completed*	XRD-0394 + palliative RT in AST

NCI, National Cancer Institute. peposertib, BY101298, XRD-0394, DNA-PK inhibitors under investigation. Tuvusertib, ATR inhibitor; AST, advanced solid tumors; CAIX, Carbonic Anhydrase IX; 177Lu-TLX250, lutetium 177- labelled anti-carbonic anhydrase IX monoclonal antibody; RT, radiotherapy; Avelumab, anti-PD-L1 monoclonal antibody.

## KRAS G12D as a therapeutic target in PDAC

KRAS mutations are a critical driver of PDAC oncogenesis and a key determinant of treatment response. KRAS functions as a GTPase with a binary switch: GDP-bound KRAS is inactive while guanine nucleotide exchange factor (GEF)-mediated exchange for GTP activates it. KRAS then modulates pathways for cellular proliferation, such as the PI3K/Akt/mTOR and RAF/MEK/ERK pathways. Both the GTPase-activating proteins (GAPs) and the intrinsic KRAS GTPase hydrolyze GTP to GDP, converting KRAS back to its inactive state (57, 128).

KRAS mutations have been found in approximately 90% of PDAC (57). The glycine-to-aspartate mutation at codon 12 (G12D) confers resistance against GTP hydrolysis, creating a constitutively activated KRAS, which signals continuous cellular proliferation (128). This is the most common KRAS mutation in PDAC, seen in up to 45% of PDAC cases with a KRAS mutation (57). Patients with KRAS mutations have a worse prognosis than patients with wild-type KRAS (57, 129).

While KRAS is not technically a component of the DDR, KRAS mutations are nonetheless implicated in DDR dynamics. Constitutively activated KRAS not only drives unchecked cellular proliferation, but preclinical evidence suggests that it may perpetuate a “hyperactive” DDR, allowing tumor cells to evade the effects of RT (130). For example, KRAS mutants exposed to radiation show increased NHEJ and reduced mitotic catastrophe (130). Inhibiting downstream KRAS substrates can radiosensitize KRAS-mutant PDAC cell models in a DDR-dependent manner (131). Thus, targeting KRAS mutants may not only represent an independent strategy for systemic treatment but may also potentiate the effects of RT, particularly given the crosstalk between KRAS downstream signaling and DDR pathways. However, direct evidence to support the link between KRAS, RT, and the DDR pathways remains limited, underscoring the need for additional clarification.

Therapies targeting KRAS G12D have been difficult to develop, but efforts are ongoing. Of the completed clinical trials, ELI-002 is a novel cancer vaccine developed by Elicio Therapeutics that was tested in a phase I trial in PDAC and CRC patients harboring KRAS G12D or G12R. This vaccine, composed of amphiphile (Amph) mKRAS peptides and Amph-CpG immunostimulatory adjuvant antigens, accumulates in lymph nodes to stimulate a robust T-cell response against the KRAS mutants. 84% of patients demonstrated T-cell responses to mKRAS, and 24% developed biomarker clearance. Patients with T-cell responses above the median demonstrated statistically significant KRAS tumor biomarker reduction (-76% vs. -10.2%, p < 0.0014) and longer relapse-free survival (hazard ratio = 0.14 for high responders, p = 0.0167) (132). There are numerous other KRAS G12D-targeting drugs and immunotherapies under investigation in clinical trials, as reported in **Table 5**.

**Table 5:** Ongoing clinical trials of KRAS G12D-targeted therapies in PDAC.

Trial ID	Sponsor	Phase	Status	Intervention
NCT05382559	Astellas Pharma Inc	I	recruiting	ASP3082 in advanced solid malignancies with KRAS G12D
NCT06364696	Astellas Pharma Inc	I	recruiting	ASP4396 in KRAS G12D-mutated AST
NCT06040541	Revolution Medicines, Inc	I/Ib	recruiting	RMC-9805 monotherapy or dual therapy with RMC-6236 in advanced solid tumors with KRAS G12D mutation
NCT05379985	Revolution Medicines, Inc	I/Ib	recruiting	RMC-6236 in advanced solid tumors with KRAS G12 or other RAS mutation
NCT06625320 (RASolute302)	Revolution Medicines, Inc	III	recruiting	RMC-6236 vs. standard of care chemotherapy in pretreated mPDAC
NCT06385925	Tyligand Pharmaceuticals (Suzhou) Limited	I/II	recruiting	TSN1611 with KRAS G12D-mutated AST
NCT07026916	Genfleet Therapeutics (Shanghai) Inc.	II	not yet recruiting	GFH375 in KRAS G12D-mutated mPDAC
NCT06770452	Zhejiang University	II	recruiting	HRS-4642, nimotuzumab, and GEMNabP in untreated mPDAC
NCT06403735	Qilu Pharmaceutical Co., Ltd.	I	recruiting	QLC1101 in AST with KRAS G12D
NCT07020221	Verastem, Inc.	I/IIa	recruiting	VS-7375 monotherapy or with cetuximab in AST with KRAS G12D
NCT06818812	Incyte Corporation	I	recruiting	INCB186748 ± chemotherapy in AST with KRAS G12D
NCT05846516 (KISIMA-02)	Amal Therapeutics	Ib	recruiting	KISIMA-02 ± ezabenlimab in PDAC with KRAS G12D/G12V
NCT06179160	Incyte Corporation	I	recruiting	INCB161734 ± chemotherapy in AST with KRAS G12D
NCT05726864 (AMPLIFY-7P)	Elicio Therapeutics	I/II	active, not recruiting	adjuvant ELI-002 immunotherapy in NRAS or KRAS mutated solid tumors
NCT06917079	TheRas, Inc., d/b/a BBOT (BridgeBio Oncology Therapeutics)	Ia/Ib	recruiting	BBO-11818 in AST with KRAS mutations
NCT06487377	Shanghai Pudong Hospital	I	recruiting	IX001 TCR-T in KRAS-mutated pancreatic and colorectal cancer.
NCT07023731	Arvinas Inc	I/II	recruiting	ARV-806 in KRAS mutated AST
NCT06586515 (MOONRAY-01)	Eli Lilly and Company	Ia/Ib	recruiting	LY3962673 ± chemotherapy in AST harboring KRAS G12D
NCT06218914	AstraZeneca	I	recruiting	NT-112 and AZD0240 TCR-T in AST with KRAS G12D
NCT03953235	Gritstone bio, Inc.	I/II	rompleted*	SLATEv1 + ipilimumab + nivolumab in advanced metastatic solid malignancies

ASP3082, ASP4396, ARV-806, KRAS G12D degrading agents; RMC-9805, KRAS G12D RAS(ON) inhibitor; RMC-6236, RAS MULTI (ON) inhibitor; TSN1611, GFH375, HRS-4642, QLC1101, VS-7375, INCB186748, BBO-11818, LY3962673, small molecule KRAS G12D inhibitors; AST, advanced solid tumors; mPDAC, metastatic PDAC; nimotuzumab, cetuximab, anti-epidermal growth factor receptor (EGFR) monoclonal antibody; GEMNabP, gemcitabine + nanoparticle albumin–bound paclitaxel (nab-paclitaxel); KISIMA-02, peptide immune-stimulatory cancer vaccine; ezabenlimab, nivolumab, PD-1 targeting monoclonal antibodies; ELI-002= lymph node-directed immunotherapy inducing T-cell responses against mutated KRAS; IXR001 TCR-T, T-cell receptor-engineered T cells targeting KRAS mutations; NT-112, AZD0240, TCR-T therapies targeting KRAS G12D; SLATEv1, neoantigen-targeting cancer vaccine; ipilimumab, CTLA-4-targeting monoclonal antibody. *Study completed, but only interim results published.

## Discussion

Radiation therapy benefits nearly half of all cancer patients but is constrained by tumor radioresistance and normal tissue toxicity. In PDAC, among the most radioresistant malignancies, radiogenomics tools such as RSI and GARD offer a framework for personalized dosing. Early studies suggest potential utility, but results remain inconsistent, signifying the need for large-scale validation and integration of radiosensitizers targeting DDR pathways (34, 39, 40).

The clinical use of the PARP inhibitor olaparib in BRCA1/2-mutated tumors demonstrates how DDR defects can be therapeutically exploited. Preclinical data support its role as a radiosensitizer in PDAC (84, 85), and an ongoing trial (NCT05411094) is testing its combination with RT in unresectable disease. Beyond PARP, inhibition of DDR components has shown radiosensitizing activity preclinically and warrants evaluation in prospective PDAC-specific trials. For example, targeting the BRCA2-RAD51 axis or inhibiting RAD52-mediated backup repair could expand synthetic lethality-based approaches beyond current applications and, importantly, potentially overcome PARP inhibitor resistance.

Importantly, combining DDR inhibition with RT may also enhance responses to immunotherapy. Radiation promotes antigen release, immune activation, and immune cell infiltration (133), while DDR inhibition increases tumor immunogenicity, type I interferon signaling, and PD-L1 expression (124, 134–136). However, these therapeutic approaches are constrained by the desmoplastic, hypovascular PDAC microenvironment, which is highly immunosuppressive and hypoxic, impairing drug delivery and promoting resistance to treatments (137, 138). Early studies of PARP inhibitors with checkpoint blockade in BRCA-mutated tumors show encouraging results (139), suggesting that rational triplet regimens (RT, DDR inhibition, immunotherapy) merit investigation. Additional strategies may include selective tumor microenvironment reprogramming, such as stromal HIF2- targeting approaches, which have shown promising results in preclinical studies (140).

As precision oncology advances, a uniform RT approach for PDAC is no longer sufficient. Prospective validation of RSI and GARD, coupled with integration of multi-omics data, will be critical to refine predictive models and personalize treatment. With the advent of biomarker-driven therapies, special considerations for trial design are also necessary. In the context of DDR inhibitor research, it is important to distinguish between well-validated DDR targets and those that are still exploratory (141). Assessment of functional pathway losses should be prioritized over simple genotypic mutation analyses alone (142, 143). Appropriate disease-specific patient stratification should be incorporated, and thorough monitoring of GI toxicities in PDAC, including acute and late effects, should be studied. Finally, study endpoints should be expanded to include serial circulating tumor (ctDNA) monitoring (144), locoregional control (145), and translational evidence of target engagement (146).

## Conclusion

PDAC remains one of the most radioresistant cancers, limiting the efficacy of conventional RT. Radiogenomics (RSI, GARD) provides a foundation for individualized dosing but lacks consistent validation. Only a few studies have combined RT with DDR inhibitors, and just three are PDAC-specific, signifying a major translational gap.

The approval of olaparib in BRCA-mutated PDAC illustrates how DDR vulnerabilities can be leveraged, and ongoing PARP–RT trials in PDAC underscore the promise of this approach. Future priorities include biomarker-driven trials testing PARP, ATR, ATM, DNA-PK, and Wee1 inhibitors with RT, as well as rational triplet regimens that incorporate immunotherapy. Integration of radiogenomics with multi-omics data and prospective validation of predictive indices will be key to guiding patient selection. Collectively, these strategies offer a path to overcoming intrinsic radioresistance and improving survival in PDAC.
